# Source Profile Analysis of Atmospheric Volatile Organic Compounds in Chongqing

**DOI:** 10.3390/toxics14020143

**Published:** 2026-02-01

**Authors:** Anqi Zhang, Xin Qi, Yuchun Jiang, Hanfei Zuo, Yang Chen, Xiaoqian Li

**Affiliations:** 1 State Key Laboratory of Environmental Criteria and Risk Assessment, Chinese Research Academy of Environmental Sciences, Beijing 100012, China; a342254602@163.com (A.Z.); midsummery@foxmail.com (Y.J.); 2 Chongqing University, Chongqing 400044, China; qixin@cigit.ac.cn; 3 Research Center for Atmospheric Environment, Chongqing Institute of Green and Intelligent Technology, Chinese Academy of Sciences, Chongqing 400714, China; chenyang@cigit.ac.cn; 4 Tianfu Yongxing Laboratory, Chengdu 610213, China; 5 Zhejiang Provincial Key Laboratory of Organic Pollution Process and Control, College of Environmental and Resource Sciences, Zhejiang University, Hangzhou 310058, China; 12414029@zju.edu.cn

**Keywords:** VOCs, anthropogenic sources, industrial emissions, source profile analysis, risk assessment

## Abstract

This study presents the first systematic investigation of Volatile Organic Compounds (VOC) source profiles from key industrial sectors in Chongqing, China. Source-specific emission data were collected from fifteen representative facilities encompassing furniture manufacturing, automobile production, and chemical industries through a combination of on-site sampling and comprehensive literature review. Our results reveal distinct chemical signatures and regional variations among different source categories: furniture manufacturing emissions are dominated by alkanes (65%), chemical industries exhibit 51% alkane contribution, while automobile manufacturing demonstrates a remarkably high aromatic hydrocarbon content (64%), significantly exceeding other investigated sectors. Notably, aromatic hydrocarbons—particularly benzene derivatives—emitted from automotive manufacturing facilities pose potential carcinogenic and chronic health risks to both occupational workers and surrounding populations, necessitating prioritized regulatory intervention. These locally derived emission profiles fill a critical knowledge gap in regional VOC source characterization for Chongqing, providing essential scientific evidence for accurate source apportionment and formulation of sector-specific emission reduction strategies.

## 1. Introduction

Volatile Organic Compounds (VOCs) are defined as all organic chemicals exhibiting a saturated vapor pressure ≥ 70 Pa at ambient temperature and a boiling-point range of 50–260 °C at standard pressure. These species actively participate in the photochemical formation of tropospheric ozone (O_3_) and secondary organic aerosols (SOAs), exerting a decisive influence on regional O_3_ episodes and PM_2.5_ pollution, and constitute key precursors of urban haze and photochemical smog [1]. Most VOCs possess unpleasant odors and exhibit toxicity, irritation, teratogenicity, and carcinogenicity; benzene, toluene, and formaldehyde, in particular, impose substantial adverse health effects [2,3,4]. Owing to their structural diversity, VOCs are conventionally classified into six chemical families: alkanes, alkenes, alkynes, aromatics, halogenated hydrocarbons, and oxygenated VOCs (OVOCs) [5]. Individual VOCs differ markedly in their physicochemical properties, such as ozone-formation potential and health hazard. Numerous studies have quantified carcinogenic and non-carcinogenic risks, demonstrating that aromatics, aldehydes, and halogenated hydrocarbons exert varying degrees of harm to human health [6,7,8,9]. Consequently, accurate characterization of VOC speciation from distinct emission sources and comprehensive toxic-risk assessment are prerequisite steps toward establishing a robust VOC source-profile database, indispensable for O_3_ source apportionment and effective pollution mitigation.

VOCs sources are broadly categorized as biogenic (BVOCs) and anthropogenic (AVOCs) [10]. BVOCs are dominated by vegetation emissions [11], whereas AVOCs arise from stationary combustion [12], mobile sources [13], industrial processes [14], solvent use [15], fuel storage and transportation [16], biomass burning [17], cooking fumes [18], solid-waste treatment [19], and miscellaneous anthropogenic activities [20].

To date, most VOC studies report ambient concentrations; speciated characterization of individual emission sources—especially key industrial sectors—remains scarce. Nationwide, industrial VOC emissions increased 11.6-fold between 1980 and 2010 [21], underscoring that abating industrial sources is pivotal to curbing China’s total VOC burden. Chongqing exemplifies this challenge: its industrial structure is highly heterogeneous, emission nodes are numerous, and VOC fingerprints differ markedly among sectors, rendering source-profile construction particularly demanding. Moreover, anthropogenic VOCs pose non-negligible health risks [22,23,24]. To quantify sector-specific emissions and toxic impacts in Chongqing, we selected representative industrial parks and conducted in-plant measurements along the full production chains of typical facilities. By documenting raw materials, products, process configurations, and emission behaviors, we provide robust data for evaluating industrial VOC emissions in Chongqing and across China, and help identify the priority enterprises for control.

## 2. Materials and Methods

### 2.1. Sampling Period and Sites

Industrial parks were targeted to capture VOC emissions from key processes. Fifteen facilities were selected in Chongqing, covering furniture manufacturing, automobile production, and the chemical industry. On-site investigations documented raw materials, products, process configurations, and emission characteristics. Stack sampling was performed at each facility between May and July 2020; details are provided in Table 1.

### 2.2. Sampling Method

The 8 h integrated samples were collected at each sampling location. Whole-air samples were in pre-cleaned, evacuated SUMMA canisters. After removing the protective nut and plug, a 2 µm PTFE filter was attached and inserted as deeply as possible into the stack. For ducts under negative pressure, the annulus between the probe and the port was sealed with a PTFE gasket to prevent ambient ingress. The canister valve was then opened to start sampling; after ~2 min, the valve was closed clockwise, the filter removed, and the transport plug reinstalled.

### 2.3. Analytical Method

Samples were analyzed with a TH300B–GC–FID/MS system (Wuhan Tianhong Instruments Co., Ltd., Wuhan, China). Sample pre-concentration was performed with a TH300B unit (Wuhan Tianhong Instruments Co., Ltd., Wuhan, China), and separation/identification with an Agilent 7820A-5977E GC-MS (Agilent Technologies, Inc., Santa Clara, CA, USA). Both instruments are verified by metrological certification; certificate details are given in Table 2.

The TH300B-GC-FID/MS system used an ultra-low-temperature empty-tube trap at −160 °C, followed by thermal-desorption injection to complete one enrichment–desorption–analysis–back-flush cycle per hour. C_2_–C_5_ hydrocarbons were separated on a PLOT Al_2_O_3_/KCl column and detected by FID (channel I), while C_5_–C_10_ hydrocarbons, halocarbons, and oxygenated VOCs were separated on a DB-624 column and detected by MS (channel II). Overall detection limits ranged from 20 ppt to 300 ppt (≈0.08–1.2 µg m^−3^ for benzene). Quantitative repeatability was ≤15% RSD, linearity error ≤ ±10% (r ≥ 0.995), and expanded uncertainty ≈ 25% (k = 2), meeting the requirements for online VOC monitoring.

A total of 57 target compounds were quantified, as listed in Table 3.

## 3. Results

### 3.1. VOC Source Profiles in Chongqing

**Figure 1:**
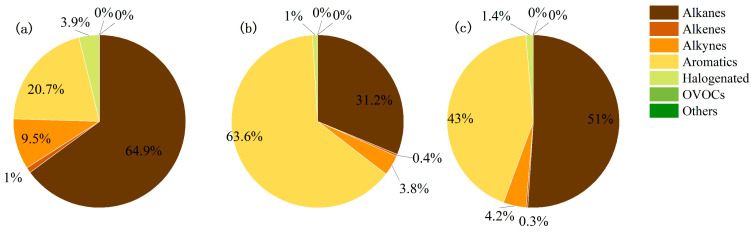
Mean fractional composition of VOC classes in the source profiles of the major AVOC emitters in Chongqing ((**a**) represents furniture manufacturing, (**b**) represents automotive manufacturing, and (**c**) represents the chemical industry, which are three types of VOC emission sources).

Figure 1 summarizes the speciated profiles for the three investigated sectors. In furniture manufacturing, alkanes dominated (65%), followed by aromatics (21%) and alkenes (9%); alkynes and halocarbons contributed 4% and 1%, respectively. Vehicle-coating operations were characterized by an aromatic-rich signature (64%), with alkanes accounting for 31% and alkenes 4%; alkynes represented 1%. Chemical-industry emissions were more evenly split between alkanes (51%) and aromatics (43%), while alkenes and alkynes comprised 4% and 2%.

### 3.2. Regional Discrepancies in Typical Anthropogenic Source Profiles

To quantify inter-city variability, we compared VOC fingerprints of the same industries reported for different regions, restricting the analysis to profiles that speciate ≥90% of the emitted mass. Here we first address furniture manufacturing and vehicle-coating emissions in Chongqing, followed by a sector-specific examination of several chemical-industry sources.

#### 3.2.1. Furniture Manufacturing

We compared the solvent-use VOC profile measured in Chongqing with the recently published Guangdong inventory [25]. Both datasets show a qualitatively similar pattern—alkanes and aromatics dominate—but quantitative splits differ. Alkanes contributed 13–65% and aromatics 21% in the two cities, while the sum of m,p-xylene, toluene, ethyl-benzene, o-xylene, and styrene consistently accounted for 8–17% of total mass, identifying these five species as robust tracers for furniture solvent emissions (Table 4).

**Figure 2:**
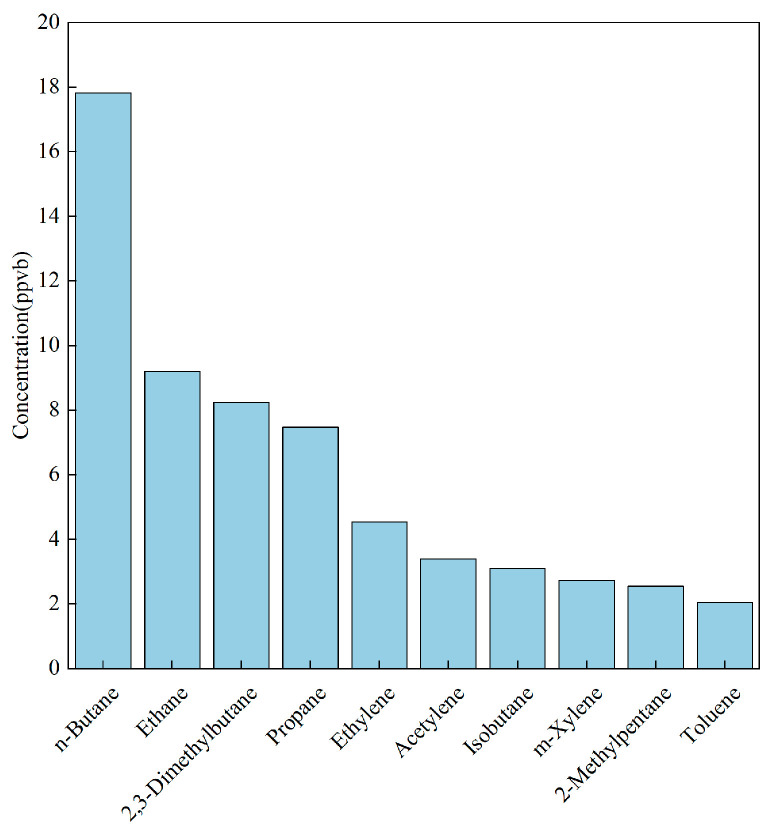
Main VOC species emitted by the furniture manufacturing industry in Chongqing.

Region-specific disparities are nevertheless evident. In Chongqing, alkanes are markedly enriched: n-butane, ethane, 2,3-dimethyl-butane, and propane together comprise 20–8% of the profile (Figure 2), almost twice their collective share in Guangdong. Conversely, Guangdong exhibits a pronounced oxygenated fraction (OVOCs ≈ 30%), underscoring the influence of regional factors on source-profile composition.

#### 3.2.2. Vehicle Manufacturing

Solvent-use VOC profiles were established for three automobile plants in Chongqing and one in Shanghai [26]. Except for Chongqing I, aromatics dominated at all facilities, accounting for 67–99% of the speciated mass; Chongqing I was uniquely alkane-rich.

As shown in Table 5, isopentane alone represented 34% of Chongqing I’s emissions, with n-pentane, isobutane, and n-butane also abundant. Aromatics ranked second, led by toluene (19%) and m,p-xylene (8%).

**Figure 3:**
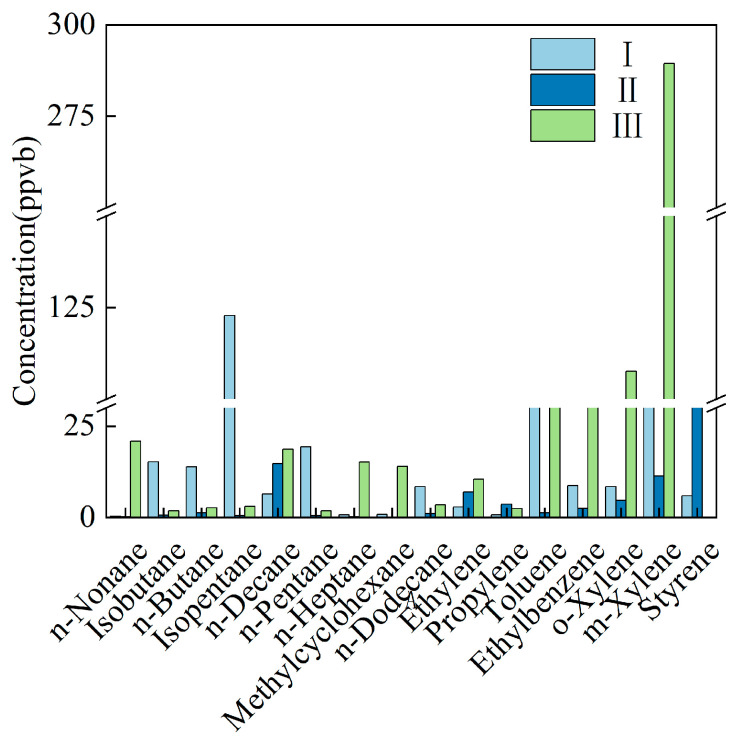
Main VOC species emitted by the vehicle manufacturing industry in Chongqing.

Chongqing II displayed the highest aromatic fraction; the top five species were m,p-xylene, styrene, n-decane, o-xylene, and ethyl-benzene. Chongqing III was almost pure styrene (97%), with only 1% m,p-xylene (Figure 3).

#### 3.2.3. Industrial Processes

**Figure 4:**
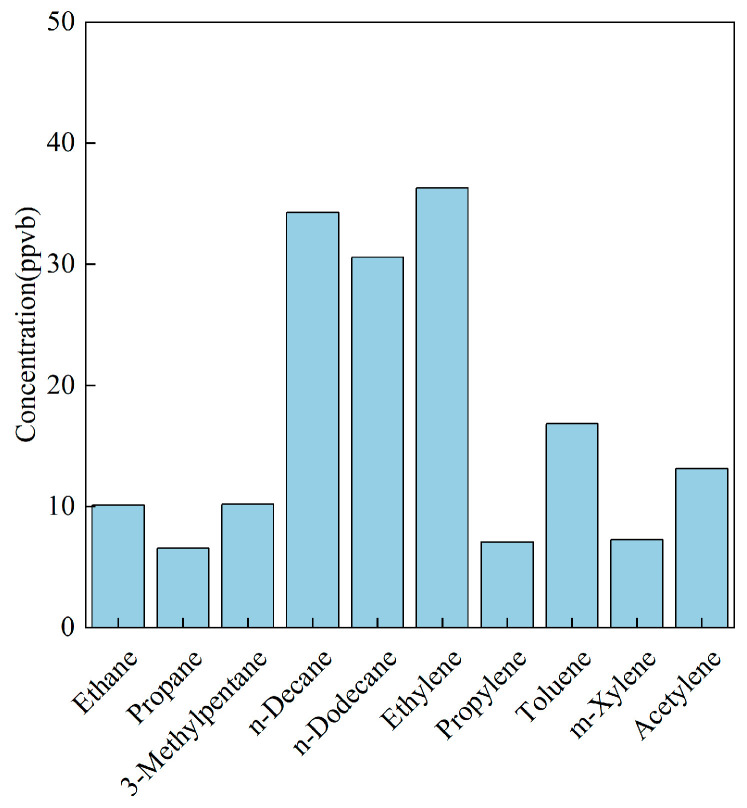
Main VOC species emitted by chemical process sources in Chongqing.

One Chongqing-based chemical-process profile (tire-rubber internal mixing) was compared with a recent dataset from the Pearl River Delta [27]. For the Chongqing facility, alkanes dominated (≈60%), followed by aromatics; the top five species were ethene, n-decane, n-dodecane, toluene, and acetylene (Figure 4). In contrast, the PRD chemical plant was aromatic-rich (≈50%) with OVOCs as the second-largest class (Table 6). These contrasts highlight the strong sector- and process-dependence of VOC fingerprints even within the same industrial umbrella.

#### 3.2.4. Petrochemical Industry

**Figure 5:**
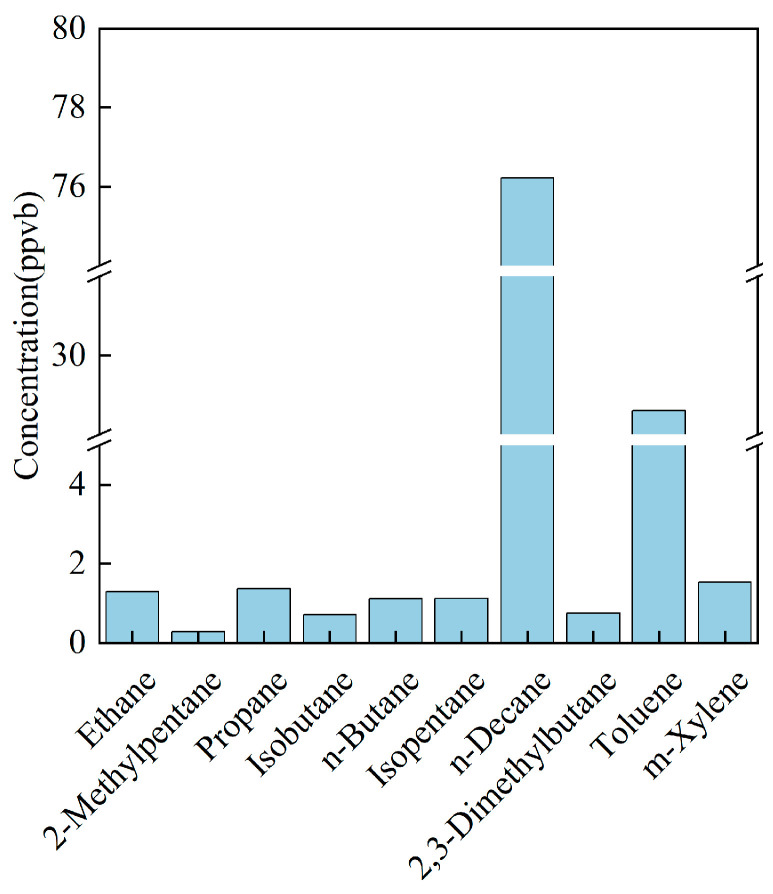
Main VOC species emitted by petrochemical sources in Chongqing.

Two petrochemical source profiles—one from Chongqing and one from Beijing [28]—were examined (Table 7). Both are alkane-dominated, with aromatics as the second-largest class, yet the dominant homologues differ by site. In the Chongqing lubricant-oil unit, n-decane alone accounted for 64% of the speciated mass, followed by toluene at 24% (Figure 5), illustrating that even within the same sector, feedstock and unit operation choices create distinct VOC fingerprints. Some studies have shown that ethylene, propylene, benzene, toluene, and styrene exhibit the highest factor loadings in petrochemical sources [29].

#### 3.2.5. Solvent Use

**Figure 6:**
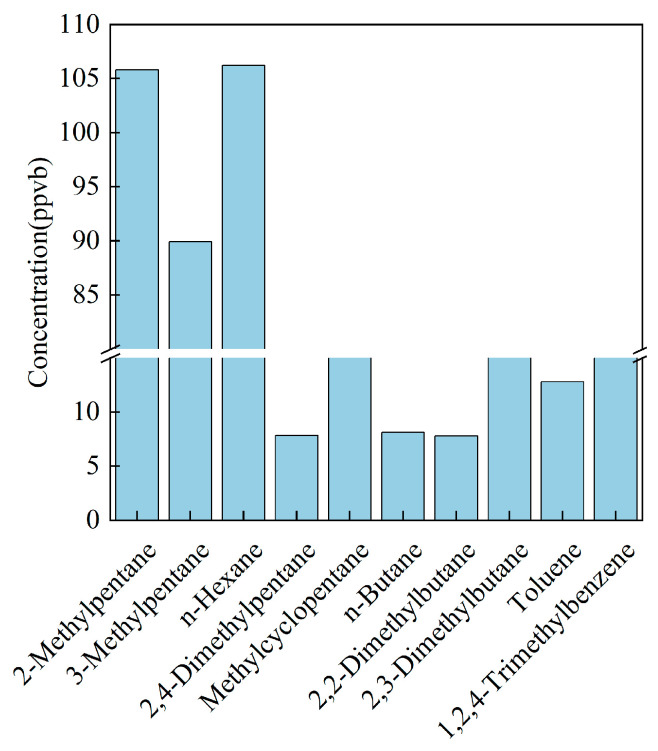
Main VOC species emitted by the electronic equipment industry in Chongqing.

Two solvent-use profiles were compiled: one for an electronics plant in Chongqing and one for a coating-solvent facility in Zhejiang (Table 8) [30]. In the Chongqing electronics source, alkanes prevailed (≈55%); n-hexane, 2-methylpentane, 3-methylpentane, methyl cyclopentane, and 2,3-dimethylbutane ranked as the top five species, whereas aromatics were limited to 1,2,4-trimethylbenzene and toluene (Figure 6). Conversely, the Shaoxing (Zhejiang) electronics stack was halocarbon-rich (31.7%), with alkanes contributing 30.4%. These large discrepancies confirm that solvent-use fingerprints are dictated by the exact thinner blends employed in each sector. Measurements of solvent-based and water-borne coatings show that highly reactive species—such as aromatic hydrocarbons and oxygenated VOCs—dominate the emissions [31], whereas printing processes are characterized primarily by long-chain alkanes and heavy aromatic hydrocarbons [32].

#### 3.2.6. Metal Spraying

Three Chongqing profiles for metal-surface coating and one Wuhan profile for automotive repair spraying [33] were compared. Except for Chongqing III, aromatics dominated the two remaining Chongqing stacks, whereas the Wuhan source was richest in halogenated hydrocarbons in Table 9.

**Figure 7:**
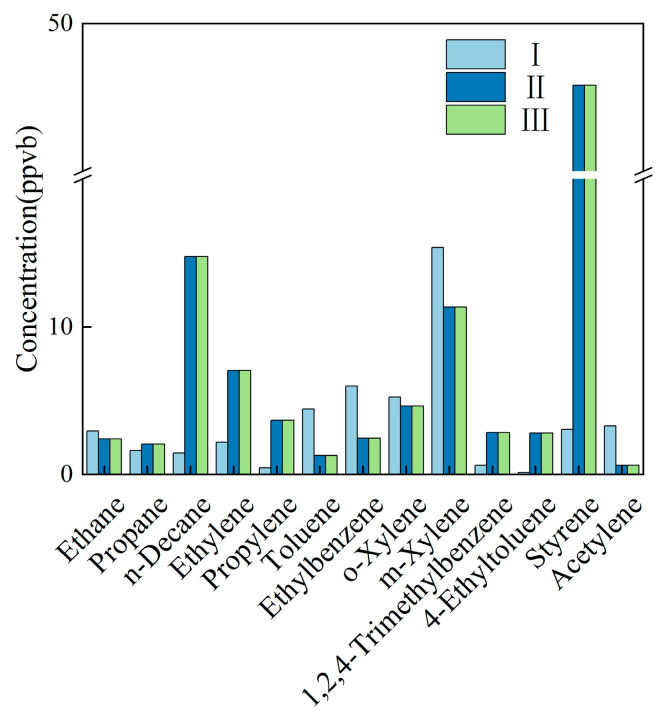
Main VOC species emitted by the metal surface coating industry in Chongqing.

The Chongqing I and Chongqing II fingerprints were broadly similar: the top six species were m,p-xylene, ethyl-benzene, o-xylene, toluene, acetylene, and styrene for I, and styrene, n-decane, m,p-xylene, ethene, o-xylene, and propene for II. Chongqing III’s profile was instead alkene-dominated, with ethene and propene alone accounting for 69.3% and 17.6% of the total mass, respectively. The main VOCs emitted from solvent-based coating/spraying processes primarily include alcohols, benzenes, esters, and aldehydes [32] (Figure 7).

#### 3.2.7. Plastic-Product Manufacturing

The database contains three solvent-use profiles from Chongqing plastic plants and one from the Yangtze River Delta [34]. Table 10 shows that Chongqing sources are alkane-dominated, with ethane alone exceeding 20%, whereas the Yangtze Delta profile is richest in halogenated hydrocarbons.

**Figure 8:**
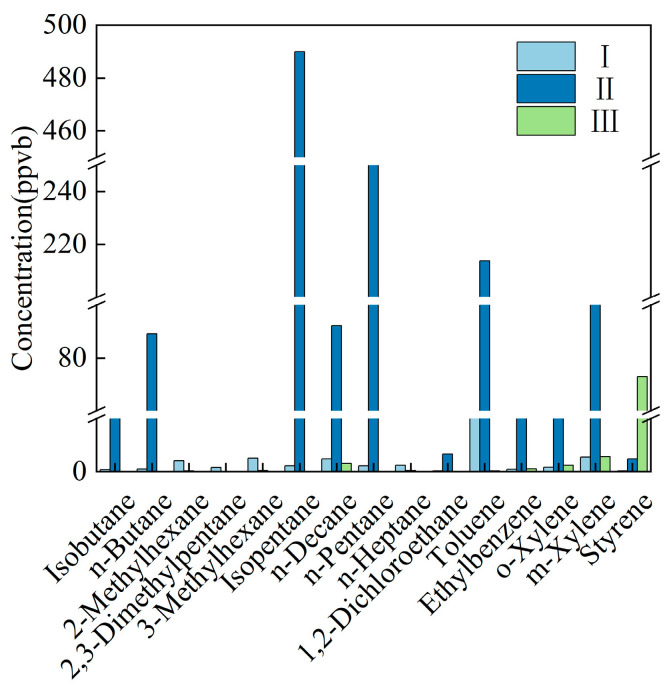
Main VOC species emitted by the plastic products manufacturing industry in Chongqing.

Within Chongqing, I and II share similar fingerprints: toluene, m,p-xylene, and n-decane all appear among the top five species. Toluene represents 34% of Chongqing I’s emissions, while isopentane (31%) and n-pentane (19.7%) head the list at Qingling (Figure 8). Chongqing III’s profile is instead aromatic-rich, with styrene reaching 83% of the total mass.

## 4. Discussion

The VOC profile of the furniture manufacturing sector is dominated by alkanes (volume fraction 65%), primarily originating from the extensive use of C6–C12 straight- and branched-chain alkanes in solvent-borne coatings, adhesives, and wood preservatives [34]. In contrast, aromatics account for 64% of the total VOC emissions from automobile manufacturing, a pattern directly linked to the widespread use of toluene, xylene, and trimethylbenzene in basecoats and clearcoats. The chemical industry exhibits a bimodal “alkane–aromatic” distribution (51% vs. 43%), reflecting integrated processes that consume naphtha and liquefied petroleum gas while simultaneously producing benzene-based intermediates. These three source profiles are statistically distinct and can serve as robust chemical fingerprints in receptor-oriented source apportionment models.

Further analysis of benzene, toluene, and xylene (BTX) reveals that their combined contributions reach ~60% of total VOCs in both automotive and chemical plant emissions, significantly exceeding the 20% observed for furniture manufacturing. Owing to their high acute toxicity and carcinogenic potential, together with secondary formation of toxic carbonyls such as formaldehyde and glyoxal during atmospheric oxidation, BTX emissions pose chronic health risks to occupational workers and residents within 1–3 km of the facilities [35,36]. Consequently, priority should be given to monitoring and controlling benzene-series compounds released by the automotive and chemical industries to achieve precise regulation of high-risk VOCs.

The close linkage between solvent recipe and emitted spectrum confirms that the above five aromatics can serve as chemical fingerprints in receptor modeling [37,38]. Their elevated abundance relative to other anthropogenic sectors makes them priority markers for attributing ambient VOCs to furniture-manufacturing activities and for designing solvent-substitution control policies.

In the field of vehicle manufacturing, across the sector, n-/iso-butanes and -pentanes, higher n-alkanes, toluene, xylenes, trimethyl-benzenes, ethyl-toluenes, together with the OVOCs n-butyl acetate and propylene-glycol methyl-ether acetate and light alcohols, are consistently elevated [39,40]. Their exact distribution is, however, tightly linked to paint formulation, application technology, and abatement configuration.

Across the results of industrial processes, the tire-compounding workshop in Chongqing displays a “dominated-by-alkanes, followed-by-aromatics” emission pattern, whereas the same process in the Pearl River Delta (PRD) shows aromatics as the largest contributor and OVOCs as the second, with alkanes markedly reduced. This inter-regional discrepancy demonstrates that raw-material formulation, process temperature, and tail-gas control level exert a decisive influence on the type of VOCs released.

In summary, VOC source profiles from China’s petrochemical industry are commonly dominated by alkanes, followed by aromatics, yet notable plant-to-plant and region-to-region disparities exist. Consequently, establishing localized profiles and selecting the species with the highest factor loadings as tracers are essential for accurately identifying and quantifying petrochemical VOC emissions.

Although electronic-device and coating-solvent emissions are conventionally grouped under the umbrella of “solvent evaporation,” their dominant VOC categories diverge markedly once industry, process, and region are taken into account. Coating-solvent profiles shift sharply with product type: solvent-borne and water-borne coatings are dominated by highly reactive aromatics and oxygenated VOCs, whereas printing operations are characterized by long-chain alkanes and heavy aromatics. These contrasts underscore that solvent use cannot be treated as a single source type; instead, high-resolution, locality-specific source profiles must be established for each clearly defined industrial process.

Based on the comprehensive source profiles of metal surface coatings in Chongqing and Wuhan, this source category is jointly regulated by three factors: enterprise, region, and coating type. It exhibits a trend dominated by aromatic hydrocarbons, with high levels of halogenated hydrocarbons and prominent alkenes. In Chongqing, Enterprises I and II share highly similar profiles, with benzene series such as m/p-xylene, ethylbenzene, o-xylene, and toluene serving as core tracers (Figure 7). This reflects the common use of traditional solvent-based coatings that contain benzene-series diluents. In contrast, samples from Wuhan show halogenated hydrocarbons ranking first, suggesting the widespread use of chlorine/fluorine-based cleaning agents or special functional additives in the region. Compared to the literature-reported [31] profile of “solvent-based coatings jointly characterized by alcohols, esters, aldehydes, and benzene series,” this study further reveals that metal surface coating is not a uniform “benzene-series source.” Instead, it should be subdivided into sub-sources based on coating formulations, processes, and pretreatment agents.

The VOC source profiles for plastic product manufacturing and solvent use in this research source library indicate that VOC emissions from different regions and enterprises are jointly influenced by variations in raw material formulations and production processes. Plastic manufacturing and solvent use sources are not uniformly “alkane-based” or “benzene-series-based”. Therefore, further subdivision based on region, enterprise, production process, and raw materials is necessary to accurately support regional VOC source apportionment and control strategies.

## 5. Conclusions

Atmospheric VOC sources are highly heterogeneous; their source profiles are shaped by industry type, feedstock properties, production technologies, air-control devices, and analytical protocols. Consequently, both the chemical classes and the abundances of individual species differ markedly among source categories. Process emissions are richest in aromatics, followed by alkanes and OVOCs, whereas solvent-use sources place aromatics first, OVOCs second, and alkanes third. Even when two sources exhibit similar class-level splits, scrutiny at the species level reveals distinct fingerprints that can unambiguously separate their contributions. Meanwhile, emission sources dominated by aromatic hydrocarbons should be given priority in control measures. VOC emissions vary by region, enterprise, process, and feedstock; source profiles must be disaggregated accordingly to enable accurate attribution and targeted control. This study bridges the gap in local industrial VOC source profiles for Chongqing, furnishing place-specific scientific evidence that enables accurate source apportionment and targeted, industry-specific emission-reduction strategies.

## Figures and Tables

**Table 1:** Sampling details for major VOC-emitting industries in Chongqing.

Industrial Category	Facilities Sampled	Sampling Location	Survey Date
Furniture manufacturing	1	Exhaust stack of spray-painting booth	11 July 2020
Automobile manufacturing	4	Exhaust of paint spray booth	8 July 2020
		Coating exhaust	1 June 2020
		P29 thermal-desorption off-gas	20 May 2020
		Engine-block spray-painting line	1 June 2020
Chemical industry	10	Spray-painting room	3 June 2020
		Paint-drying oven	1 June 2020
		Stack of printed-circuit-board treatment unit	3 June 2020
		Coating exhaust outlet	20 May 2020
		Coating exhaust outlet	20 May 2020
		Exhaust from cleaning-solvent scrubber	20 May 2020
		Emission point of glue-dripping process	20 May 2020
		Ether-based oil production vent	20 May 2020
		Internal-mixer exhaust	8 July 2020
		Color-filter cell assembly exhaust	26 May 2020

**Table 2:** Metrological verification of the analytical instruments.

Item	Model/Specification	Serial No.	Traceability Date	Validity (Months)
TH300B-GC-FID/MS	7820A/5977E	CN15262016/US1526Q201	1 November 2019	12

**Table 3:** Fifty-seven target VOCs measured for the Chongqing source-profile database.

Compound Name	CAS No.	Category
Ethane	74-84-0	Alkanes
Propane	74-98-6	Alkanes
Isobutane	75-28-5	Alkanes
n-Butane	106-97-8	Alkanes
Isopentane	78-78-4	Alkanes
n-Pentane	109-66-0	Alkanes
2,2-Dimethylbutae	75-83-2	Alkanes
Cyclopentane	287-92-3	Alkanes
2,3-Dimethylbutane	79-29-8	Alkanes
2-Methylpentane	107-83-5	Alkanes
3-Methylpentane	96-14-0	Alkanes
n-Hexane	110-54-3	Alkanes
2,4-Dimethylpentane	108-08-7	Alkanes
Methylcyclopentane	96-37-7	Alkanes
Cyclohexane	110-82-7	Alkanes
2-Methylhexane	591-76-4	Alkanes
2,3-Dimethylpentane	565-59-3	Alkanes
3-Methylhexane	589-34-4	Alkanes
2,2,4-Trimethylpentane	540-84-1	Alkanes
n-Heptane	142-82-5	Alkanes
Methylcyclohexane	108-87-2	Alkanes
2,3,4-Trimethylpentane	565-75-3	Alkanes
2-Methylheptane	592-27-8	Alkanes
3-Methylheptane	589-81-1	Alkanes
n-Octane	111-65-9	Alkanes
n-Nonane	111-84-2	Alkanes
n-Decane	124-18-5	Alkanes
n-Undecane	1120-21-4	Alkanes
n-Dodecane	112-40-3	Alkanes
Ethylene	74-85-1	Alkenes
Propylene	115-07-1	Alkenes
1-Butene	106-98-9	Alkenes
cis-2-Butene	590-18-1	Alkenes
trans-2-Butene	624-64-6	Alkenes
1-Pentene	109-67-1	Alkenes
trans-2-Pentene	646-04-8	Alkenes
Isoprene	78-79-5	Alkenes
cis-2-Pentene	627-20-3	Alkenes
1-Hexene	592-41-6	Alkenes
Acetylene	74-86-2	Alkynes
1,2-Dichloroethane	107-06-2	Halogenated hydrocarbons
Benzene	71-43-2	Aromatics
Toluene	108-88-3	Aromatics
p-Xylene	106-42-3	Aromatics
Ethylbenzene	100-41-4	Aromatics
m-Xylene	108-38-3	Aromatics
Styrene	100-42-5	Aromatics
o-Xylene	95-47-6	Aromatics
Isopropylbenzene	98-82-8	Aromatics
n-Propylbenzene	103-65-1	Aromatics
o-Ethyltoluene	611-14-3	Aromatics
m-Ethyltoluene	620-14-4	Aromatics
1,3,5-Trimethylbenzene	108-67-8	Aromatics
p-Ethyltoluene	622-96-8	Aromatics
1,2,4-Trimethylbenzene	95-63-6	Aromatics
1,2,3-Trimethylbenzene	526-73-8	Aromatics
m-Diethylbenzene	141-93-5	Aromatics
p-Diethylbenzene	105-05-5	Aromatics

**Table 4:** Mass fractions (%) of individual VOC species emitted from furniture manufacturing under the solvent-use source category.

Species Category	Chongqing	Guangdong
Alkanes	64.95	13.18
Alkenes	9.48	0.00
Alkynes	3.86	0.00
Aromatics	20.68	20.73
Halogenated hydrocarbons	1.03	6.98
OVOCs	0.00	59.11

**Table 5:** Proportions of various VOCs emitted by the vehicle manufacturing industry in solvent use sources (%).

Species Category	Chongqing I	Chongqing II	Chongqing III	Shanghai
Alkanes	60.58	27.66	0.60	7.40
Alkenes	1.88	3.69	0.08	0.00
Alkynes	0.43	0.95	0.04	0.00
Aromatics	36.73	67.44	99.27	92.40
Halogenated hydrocarbons	0.38	0.25	0.01	0.00
OVOCs	0.00	0.00	0.00	0.20

**Table 6:** Proportions of individual VOC species emitted from chemical-process sources (%).

Species Category	Chongqing Tire Banbury Workshop	Pearl River Delta
Alkanes	50.03	12.00
Alkenes	17.86	0.00
Alkynes	5.11	0.00
Aromatics	26.38	47.00
Halogenated hydrocarbons	0.63	3.00
OVOCs	0.00	37.00

**Table 7:** Proportions of individual VOC species emitted from petrochemical sources.

Species Category	Chongqing Lubricant Production	Beijing
Alkanes	71.02	42.00
Alkenes	1.03	15.00
Alkynes	0.39	0.00
Aromatics	27.40	24.00
Halogenated hydrocarbons	0.17	5.00
OVOCs	0.00	12.00

**Table 8:** Proportions of individual VOC species emitted by the electronic-equipment industry within solvent-use sources (%).

Species Category	Chongqing PCB Spraying	Shaoxing, Zhejiang
Alkanes	85.47	30.40
Alkenes	0.90	3.10
Alkynes	0.48	0.00
Aromatics	12.88	23.30
Halogenated hydrocarbons	0.27	31.70
OVOCs	0.00	11.50

**Table 9:** Proportions of individual VOC species emitted from metal surface coating within solvent-use sources (%).

Species Category	Chongqing I	Chongqing II	Chongqing III	Wuhan
Alkanes	24.71	21.65	4.60	20.00
Alkenes	5.36	10.42	88.33	2.00
Alkynes	5.72	0.54	2.55	0.00
Aromatics	62.49	66.59	4.51	14.00
Halogenated hydrocarbons	1.72	0.79	0.01	70.00
OVOCs	0.00	0.00	0.00	4.00

**Table 10:** Proportions of individual VOC species emitted from plastic-product manufacturing within solvent-use sources (%).

Species Category	Chongqing I	Chongqing II	Chongqing III	Yangtze River Delta
Alkanes	46.18	66.39	4.65	20.00
Alkenes	1.66	0.20	0.47	2.60
Alkynes	0.52	0.71	0.11	0.00
Aromatics	51.19	32.30	94.77	23.00
Halogenated hydrocarbons	0.46	0.42	0.00	36.00
OVOCs	0.00	0.00	0.00	11.00

## Data Availability

The data presented in this study are available on request from the corresponding author.
